# Characterization of *LEF1* High Expression and Novel Mutations in Adult Acute Lymphoblastic Leukemia

**DOI:** 10.1371/journal.pone.0125429

**Published:** 2015-05-05

**Authors:** Xing Guo, Run Zhang, Juan Liu, Min Li, Chunhua Song, Sinisa Dovat, Jianyong Li, Zheng Ge

**Affiliations:** 1 The First Affiliated Hospital of Nanjing Medical University, Jiangsu Province Hospital, Department of Hematology, Nanjing, 210029, China; 2 Pennsylvania State University Medical College, Department of Pediatrics, Hershey, 17033, PA, United States of America; University of Heidelberg, GERMANY

## Abstract

Aberrant activation of the Wnt pathway plays a pathogenetic role in tumors and has been associated with adverse outcome in acute lymphoblastic leukemia (ALL). Lymphoid enhancer binding factor 1 (*LEF1*), a key mediator of Wnt signaling, has been linked to leukemic transformation, and *LEF1* mutations have been identified in T-ALL. Here we found *LEF1* is highly expressed in 25.0% adult ALL patients and *LEF1* high expression was associated with high-risk leukemia factors (high WBC, Philadelphia chromosome positive, complex karyotype), shorter event-free survival (EFS), and high relapse rates in patients with B-ALL. *LEF1* high expression is also associated with high mutation rate of *Notch1* and *JAK1* in T-ALL. We identified 2 novel *LEF1* mutations (K86E and P106L) in 4 of 131 patients with ALL, and those patients with high-risk ALL (high WBC, complex karyotype). These results suggest a role for *LEF1* mutations in leukemogenesis. We further explored the effect of the mutations on cell proliferation and found both mutations significantly promoted the proliferation of ALL cells. We also observed the effect of *LEF1* and its mutations on the transcription of its targets, *c-MYC* and *Cyclin D1*. We found *LEF1* increased the promoter activity of its targets *c-MYC* and *Cyclin D1*, and *LEF1* K86E and P106L mutants further significantly enhanced this effect. We also observed that the *c-MYC* and *Cyclin D1* mRNA levels were significantly increased in patients with *LEF1* high expression compared with those with low expression. Taken together, our findings indicate high *LEF1* expression and mutation are associated with high-risk leukemia and our results also revealed that *LEF1* high expression and/or gain-of-function mutations are involved in leukemogenesis of ALL.

## Introduction

Lymphoid enhancer binding factor 1 (*LEF1*), as the member of the LEF/TCF family, plays a crucial role in early lymphocyte development[1–5]. *LEF1* is normally expressed in T and pro B cells, and the knockout mice exhibit defects in pro-B cell survival and proliferation. It also mediates Wnt signaling-induced proliferation increase of pro B cells. *LEF1* expression is reported to be involved in leukemic transformation [4], and associated with poor prognosis in adult B precursor acute lymphoblastic leukemia (ALL) [6], chronic lymphocytic leukemia(CLL)[6,7], cytogenetically normal acute myeloid leukemia (AML), and adult de novo acute promyelocytic leukemia (APL)[8]. These reports indicate that *LEF1* is involved in the oncogenesis of leukemia.

The Wnt signaling pathway has been implicated in regulation of the proliferation, survival, and differentiation of hematopoietic cells. *LEF1* exerts its role in regulation of cell proliferation and survival by activation of Wnt target genes through recruiting the co-activator β-catenin [4,9]. Increased expression of *LEF1* affects normal expression of cell cycle and growth-promoting genes, such as *Cyclin D1* and *c-MYC* and disturbs differentiation in hematopoiesis [4]. Aberrant expression of *LEF1* has been reported to be involved in solid cancers and leukemia [4,10–12]; and *LEF1* is required for the growth of leukemia cells [13,14]. The increase of *LEF1* mRNA and Wnt target gene *c-MYC* was also shown in the blast phase (BP) of chronic myeloid leukemia (CML).These reports indicate that *LEF1* has an oncogenic effect by promoting cell proliferation through regulation of target gene expression. However, it is unknown if and how *LEF1* affects cell proliferation in ALL.

*LEF1* mutations have been identified and associated with high-risk events in AML and lymphoma. So far no *LEF1* mutations have been identified in B-ALL, and it is also unknown how *LEF1* mutations affect cell proliferation in ALL.

Here, we identified *LEF1* high expression in 25.0% Chinese adult ALL and identified 2 novel *LEF1* mutations in the cohort. We also found that *LEF1* high expression and novel mutations are involved in its oncogenic effect in the high-risk ALL by promoting the cell proliferation and target gene expression.

## Materials and Methods

### Patients and samples

Bone marrow (BM) samples from 131 newly diagnosed patients [82 male, 49 female; median age 34 (14–75) years old] with ALL (87 B-ALL, 43 T-ALL and 1 T-/B-ALL) were collected between June 2008 and July 2013 at the First Affiliated Hospital of Nanjing Medical University. The diagnosis of ALL was made according to the morphologic, immunophenotypic, cytogenetic, and molecular criteria of WHO Diagnosis and Classification of ALL (2008). All the patients provided their written informed consent in accordance with the Declaration of Helsinki before enrollment in the study. The study was approved by the Institutional Review Board of the Nanjing Medical University.

### Cytogenetic and molecular analyses

Conventional cytogenetic analysis was performed at the time of diagnosis, using unstimulated short-term cultures according to the recommendations of the International System for Human Cytogenetic Nomenclature (ISCN). For each sample, at least 20 bone marrow metaphase cells were analyzed.

Immunophenotypic analyses were performed by flow cytometry on fresh pretreatment BM samples. The cell-surface antigen was defined positive when fluorescence intensity of at least 20% of cells exceeded fluorescence of negative control. Determination of *BCR-ABL* was performed as previously described [15].

### *LEF1* mutation detection

We screened mutations of *LEF1* exons 2 and 3, the hotspot regions in T-and B-ALL (24, 30). Genomic DNA was isolated from pretreatment BM samples of the 131 patient cohort using QIAamp DNA Blood Mini Kit (Qiagen, USA) following the manufacturer's instructions. DNA fragments for the entire *LEF1* exons 2 and 3 were amplified by PCR using AmpliTaq Gold (Applied Biosystems) and the following primers: exon 2 forward, 5′-TTTTCTTTCTTTTGGGTGTGG; exon 2 reverse, 5′-AAATTGCACCCCTTATCTGC; exon 3 forward, 5′-AAAGGGAAGTCAGTGCATCATT; and exon 3 reverse, 5′-ACAAATCAATTTGCACTTCTGAAC. The purified PCR products were used for DNA sequencing.

### Mutational analyses of *NOTCH1*, *JAK1*, *FBXW7*, *PTEN and PHF6*

We performed mutational analyses of *JAK1* exons 13, 14, 16–19 [16], *NOTCH1* exons 26–28, 34 [17–19]. Genomic DNA was isolated following the manufacturer's instructions. DNA fragments spanning the above *JAK1* and *NOTCH1* exons were amplified by PCR using AmpliTaq Gold (Applied Biosystems) and exon-specific primers as previously reported [16, 17]. DNA sequencing was performed on purified PCR products.

We also performed mutational analysis in *FBXW7* exons 5–12 [18–20], *PTEN* exons 1–9 [21] and *PHF6* exons 2–10 [22] with the reported primers.

### Cell Culture and Reagents

Nalm6 and MOLT4 cells were obtained from American Type Culture Collection (ATCC, Manassas, VA) and cultured in RPMI 1640 medium (Cellgro) supplemented with 10% fetal bovine serum (Hyclone). HEK 293T cells were cultured in DMEM (Cellgro) supplemented with 10% fetal calf serum and 1% L-glutamine (Cellgro). Cells were incubated at 37°C in a humidified atmosphere of 5% CO_2_.

### Plasmids and site-directed mutagenesis

Human *LEF1* in Mammalian expression vector (pBABE-puro) was bought from Addgene. The *LEF1* K86E and P106L mutations were created by site-direct mutagenesis (mutagenesis kit from Stratagene) using a PCR technique, and confirmed by sequencing.

### Luciferase Assay

The pGL3 luciferase reporter constructs for promoters of *Cyclin D1 and E2F1* were purchased from addgene. The promoter of *c-MYC* (-1000bp) was cloned into pGL4.15 vector (Promega). The transient luciferase assay was performed in HEK293T cells using the Promega’ luciferase assay reagents and measured with luminometer following the manufacture’s instruction. The firefly luciferase activities were calculated as fold change relative to values obtained from pGL vector only control cells, and expressed as a percentage of pBABE-puro-LEF1 or its mutants transfection-induced luciferase activity versus that of pBABE vector. All transfection and reporter assays were performed independently, in triplicate, at least three times.

### Real Time-PCR

Total RNA was isolated using the RNeasy Mini Kit (QIAGEN). A 1 μg aliquot of RNA was reverse transcribed using a SuperScript First-Strand Synthesis System for RT-PCR Kit (Invitrogen). qRT-PCR was performed with qSTAR SYBR Master Mix (OriGene) using a StepOne Plus real-time PCR system (Applied Biosystems). Each experiment was performed in triplicate.

In order to quantitate gene expression value in patients’ samples, template standards and primers against Homo sapiens gene *LEF1* (OriGene, USA) and Homo sapiens housekeeping gene *GAPDH*(OriGene, USA) were obtained from OriGene Technologies (Rockville, MD). Gene expression values of the genes of interest (GOI) were achieved in each patient by a formula obtained with a scatter graph of the Ct values from the serial dilutions of template standard following manufacturer’s instruction and as previously reported [23]. The expression level of GOI was subsequently normalized to the housekeeping gene, expressed as gene expression value of GOI/GAPDH.

All the patients were divided into high and low *LEF1* expression groups (Q4 vs Q1-Q3) and the cut-off value (20.985) was determined by SPSS 17.0. Statistical analysis with analysis of variance (ANOVA) showed that the difference of *LEF1* expression in the two groups was very much significant (*P*<0.0001).

The *c-MYC* and *Cyclin D1* expression in patients were also quantitated similarly by the formula achieved with serial dilutions of their plasmids as template standards. The difference of their expression in the patients with high or low *LEF1* expression was statistically analyzed with ANOVA. The qPCR for *c-MYC* and *Cyclin D1* expression in the Nalm6 cells expressed with *LEF1* wide type (LEF1-WT) and its mutants were performed and the results were normalized to those obtained with 18sRNA and presented as fold induction over vector controls. Primers: *18s RNA*, Sense: 5’-GTAACCCGTTGAACCCCATT-3’, Antisense: 5’- CCATCCAATCGGTAGTAGCG-3’; *c-MYC* Sense: 5’- AATGAAAAGGCCCCCAAGGTAGTTATCC-3’, Anti-sense: 5’- GTCGTTTCCGCAACAAGTCCTCTTC-3’; *Cyclin D1* Sense: 5’- TGGTGAACAAGCTCAAGTGGA-3’, Anti-sense: 5’- GAAGGTCTGCGCGTGTTTG-3’.

### Cell proliferation assay

*LEF1*-WT, *LEF1* K86E and P106L were stably expressed in Nalm6 and Molt4 cells by puromycinselection. The colorimetric cell proliferation assay (WST-1 reagent from Roche Applied Science) was performed in 96-well white clear bottom plates (Costar) inquadruplicate, according to manufacturer’s instructions. The absorbance at 440 nm (reflects number of viable cells) was measured with a plate reader.

### Statistical analysis

Patients were divided into high and low *LEF1* expression groups (Q4 vs Q1-Q3). For quantitative parameters, overall differences between the cohorts were evaluated using a Mann—Whitney U-test. For qualitative parameters, overall group differences were analyzed using a χ2 test. Survival analysis was calculated using the Kaplan–Meier method. All statistical analyses were performed using the SPSS 17.0 and *P*<0.05 was considered statistically significant.

The experimental data are shown as the mean value with bars representing the standard error of the mean (S.E.M.). Determinations of statistical significance were performed using a Student *t*-test for comparisons of two groups or using analysis of variance (ANOVA) for comparing multiple groups. The *P*<0.05 was considered statistically significant.

## Results

### Association of *LEF1* expression with characteristics of adult ALL

**Fig 1:**
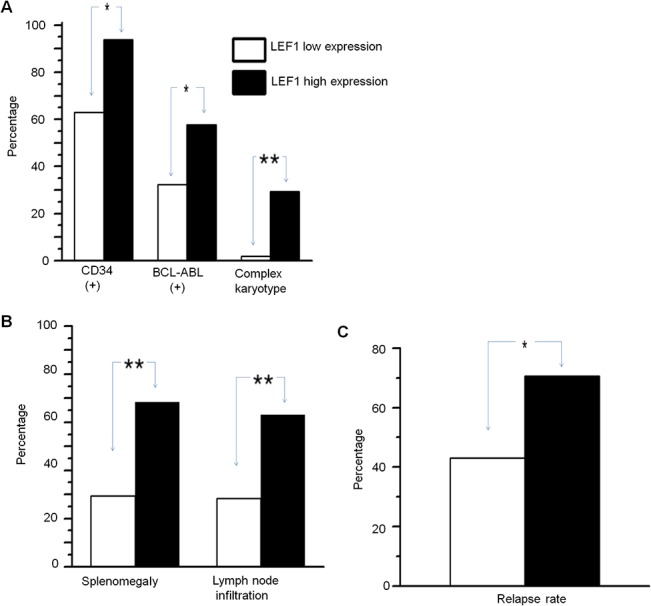
Correlation of *LEF1* high expression with clinical features in adult ALL. A-C. Association of *LEF1* expression with CD34+, BCR-ABL + and complex karyotype (A), Splenomegaly and lymph node enlargement (B) and relapse rates (C) in B-ALL. * *P*<0.05; **P<0.01.

We detected *LEF1* mRNA expression in 84 newly diagnosed adult B-ALL patients. Patients were divided into high (19) and low(65) *LEF1* expression groups. Patients with high compared to low *LEF1* expression showed higher median white blood cell counts (WBC) (126.1×10^9^/L vs 27.3×10^9^/L, *P* = 0.017), and a higher percentage of lymphoblasts in peripheral blood than those of *LEF1* low expression (87.0% vs 64.0%, *P* = 0.003) (Table 1).The patients with CD34+, BCR-ABL+/ Philadelphia chromosome positive (Ph+), or complex karyotype were significantly higher in *LEF1* high expression than those of low expression(93.8% vs 63.0%, *P* = 0.040; 57.8% vs 32.3%, *P* = 0.043; 29.4% vs 1.8%, *P* = 0.002) (Table 1, Fig 1A). The patients with *LEF1* high expression were also significantly higher for the presence of splenomegaly and lymph node enlargement compared to those with *LEF1* low expression (68.4% vs 29.4%, *P* = 0.003; 63.2% vs 28.3%, *P* = 0.007) (Table 1, Fig 1B). No significant differences in *LEF1* expression were observed with age, sex, or bone marrow blasts. These data indicated that *LEF1* high expression is associated with high-risk B-ALL.

**Table 1:** Correlation of LEF1 expression with clinical parameters in adult patients with B-ALL.

Characteristics	LEF1 Expression		P value
	Low (n = 65)	High (n = 19)	
**Age (years)**			
**Median (range)**	30.5 (14–75)	31 (14–65)	0.514
**Sex (%)**			
**male**	61.5	42.1	0.132
**WBC, ×10** ^**9**^ **/L**			
**Median (range)**	27.3 (1.5–300.0)	126.1(0.9–398.4)	0.017
**≥100×10** ^**9**^ **/L (%)**	16.1% (10/62)	44.4% (8/18)	0.027
**Blasts (%) median (range)**			
**bone marrow**	87.6 (59.0–99.0)	89.6 (64.0–100.0)	0.307
**peripheral blood**	64.0 (0–97.0)	87.0 (24.0–95.0)	0.003
**Genetics (%)**			
**BCR-ABL +**	32.3% (21/65)	57.8% (11/19)	0.043
**complex karyotype**	1.8(1/55)	29.4(5/17)	0.002
**CD34+ (%)**	63.0% (34/54)	93.8% (15/16)	0.04
**Extramedullary infiltration (%)**			
**Spleen**	29.4 (15/51)	68.4% (13/19)	0.003
**lymph node**	28.3% (15/53)	63.2% (12/19)	0.007

We also detected *LEF1* mRNA high expression in 12 of 40 newly diagnosed adult T-ALL patients. *LEF1* high expression was associated with higher median WBC and blasts in peripheral blood compared to low expression (74.0×10^9^/L vs 28.9×10^9^/L, *P* = 0.012; 76.5% vs 24.0%, *P* = 0.007). The percent of patients with mutations in *Notch1* and *JAK1* was significantly higher in the *LEF1* high expression group than the low expression group(100% vs 66.7%, *P* = 0.033; 33.3% vs 0.0%, *P* = 0.005) (Table 2), but we did not observe significant differences in mutations of *FBXW7*, *PTEN* and *PHF6*, complex karyotype or percentage of CD34+ in the two groups. The percentage of lymph node enlargement in patients with *LEF1* high expression was also significantly higher than that of low expression (100% vs 66.7%, *P* = 0.038). These data also indicated that *LEF1* high expression is associated with the unfavorable prognostic factors in T-ALL.

**Table 2:** Correlation of LEF1 expression with clinical parameters in adult patients with T-ALL.

Characteristics	LEF1 Expression		P value
	Low (n = 28)	High (n = 12)	
**Age (years)**			
**Median (range)**	36.5 (14–54)	27.5 (14–62)	0.322
**Sex (%)**			
**male**	85.7(24/28)	66.7(8/12)	0.211
**WBC, ×10** ^**9**^ **/L**			
**Median (range)**	28.9 (1.0–437.0)	74.0 (44.0–283.0)	0.012
**≥100×10** ^**9**^ **/L (%)**	26.1(6/23)	45.5(5/11)	0.434
**Blasts (%) median (range)**			
**bone marrow**	80.0 (14.0–99.0)	86.8 (56.0–98.0)	0.211
**peripheral blood**	24.0 (1.0–84.0)	76.5 (6.0–99.0)	0.007
**Genetics (%)**			
**Notch1 mutations**	66.7% (16/24)	100% (12/12)	0.033
**JAK1 mutations**	0% (0/28)	33.3% (4/12)	0.005
**FBXW7 mutations**	16.7(4/24)	8.3 (1/12)	0.646
**PTEN mutations**	15.0 (3/20)	8.3 (1/12)	1.000
**PHF6 mutations**	47.4 (9/19)	25.0 (3/12)	0.274
**complex karyotype**	4.2 (1//24)	9.1 (1/11)	0.536
**CD34+ (%)**	68.4 (13/19)	55.6 (5/9)	0.677
**Extramedullary infiltration (%)**			
**Spleen**	25.9 (7/27)	54.5 (6/11)	0.135
**lymph node**	66.7 (18/27)	100.0 (11/11)	0.038

### *LEF1* expression and outcome in adult ALL patients

**Fig 2:**
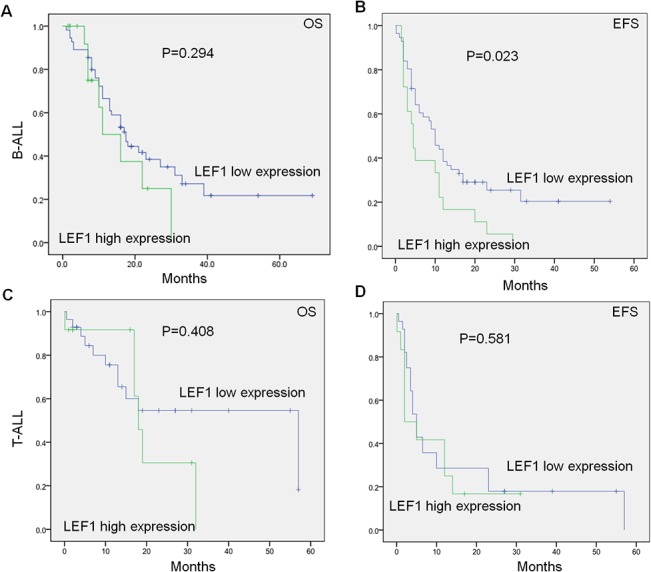
Correlation of *LEF1* expression with survival in adult ALL. A-D: *LEF1* expression with overall survival (A,C) and event-free survival (B, D) in adult B-ALL (A,B) and T-ALL(C,D).

We analyzed the *LEF1* expression with survival of the 84 B-ALL and 40 T-ALL patients. We found that B-ALL and T-ALL patients with high compared to low *LEF1* expression showed no significant differences for overall survival (OS) (11.0 months vs 17.5 months, *P* = 0.294; 18 months vs 57 months, *P* = 0.408) (Fig 2A and 2C). We also analyzed the event-free survival (EFS) in the patients and found that there was a significantly shorter EFS in B-ALL patients with *LEF1* high expression compared to low expression (4.5 months vs 10.0 months; *P* = 0.023) (Fig 2B), but no significant difference in EFS was observed in the two groups in T-ALL patients (Fig 2D). Also, we found that *LEF1* high expression in patients with B-ALL had the significantly higher relapse rate than that of low expression [70.6% (12/17) vs. 43.1% (25/58), *P* = 0.046] (Fig 1C). These results indicated that *LEF1* high expression associates with poor prognosis in B-ALL patients.

### Identification of 2 novel *LEF1* point mutations and its correlation with relapse

**Fig 3:**
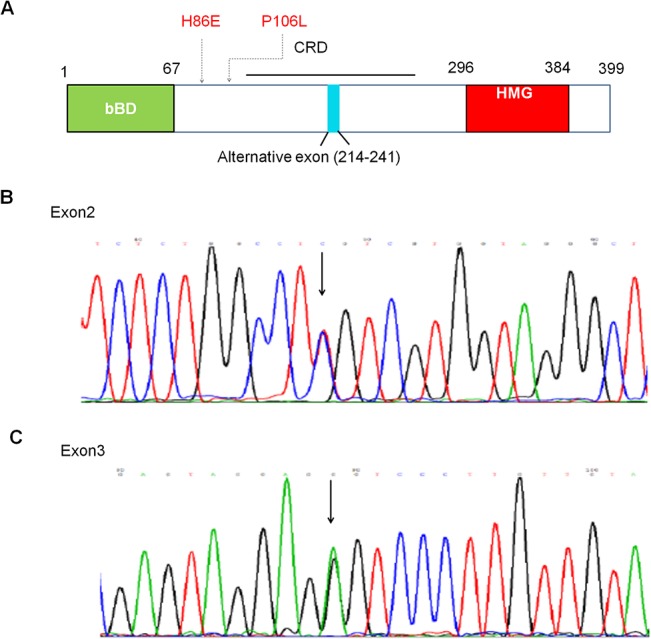
Identification of novel *LEF1* mutations in ALL patients. A. Schematic structure of the *LEF1* gene. B-C.Point mutations of *LEF1* in exon 2 (B) and exon 3 (C).

We identified 2 novel *LEF1* mutants in 4 of 131(3.1%) adult ALL patients. All are point mutations, one of them was located in exon2 and the other 3 were in exon3 (Fig 3). All 4 mutations resulted in the amino acid changes (Table 3 and Fig 3). Of the mutations, 3 were detected in B-ALL patients and 1 in T-ALL. Of the patients with *LEF1* mutants, 2 had high WBC (30.9×10^9^/L and 269.6×10^9^/L) at pretreatment. The patients with *LEF1* exon2 mutation had a complex karyotype (46,XX,t(1;3;9)(p34;p21;p21),2q+,6q-,7p-,8p-,12p-,-16,+mar[4]/46,XX). The T-ALL patient with the *LEF1* exon3 P106L mutation also had the *Notch1* exon26 mutation (L1574P). This data indicates that the patients with *LEF1* mutations also exhibited unfavorable prognostic factors. Indeed, the 2 patients with *LEF1* exon3 mutation (P106L) and high WBC relapsed in 3 months.

**Table 3:** Identification of LEF1 mutations in Chinese adult patients with ALL.

Patient ID	Exon	Nucleotide description	Protein description
ALL-s316	2	1445 C→T	K86E
ALL-s236	3	1506 G→A	P106L
ALL-s254	3	1506 G→A	P106L
ALL-s1449	3	1506 G→A	P106L

We also found *LEF1* low expression in 2 of 4 patients with *LEF1* mutation (data not shown). However, these two patients were also relapsed in 3 months, suggested that the *LEF1* mutation may have oncogenic effect.

### Effect of *LEF1* and its mutations on cell proliferation in ALL leukemia cells

**Fig 4:**
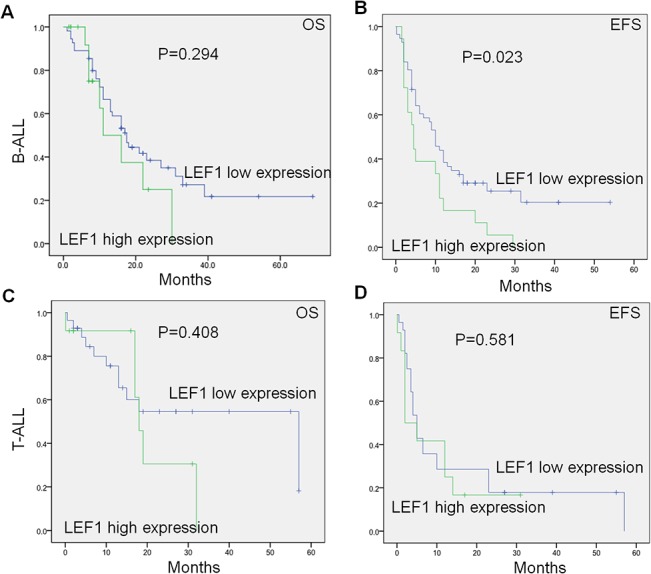
*LEF1* mutants promote cell proliferation of ALL cells. A-B.*LEF1* wild-type (*LEF1*-WT) and its mutants (*LEF1*-K86E and *LEF1*-P106L) were expressed in Molt4 (A) and Nalm6 (B) cells. * *P*<0.05; **P<0.01; ***P<0.001.

In order to explore the effect of *LEF1* and its mutations on ALL, we stably expressed *LEF1*-WT and its mutants K86E and P106L in Nalm6 B-ALL and Molt4 T-ALL leukemia cells with puromycin selection and also cells with vector only as control. We found that cell proliferation was significantly increased in *LEF1*-WT-expressed Nalm6 and Molt4 cells compared to vector only cells (Fig 4), and the cells expressing K86E or P106L significantly increased the proliferation of both Nalm6 and Molt4 cells compared to *LEF1*-WT (Fig 4). These data indicated that *LEF1* promoted ALL cell proliferation and the two novel mutants increased the stimulatory effect of *LEF1* on proliferation.

### *LEF1* and its mutations increased the transcription of *c-MYC* and *Cyclin D1*

**Fig 5:**
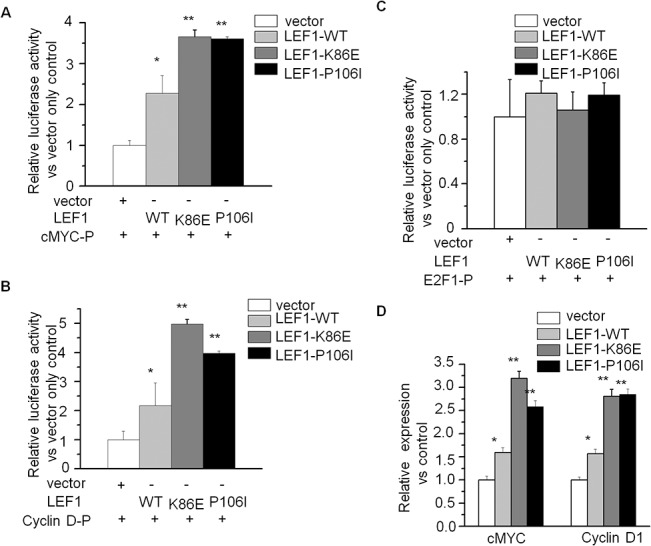
*LEF1* mutants regulate transcription of *c-MYC*, *Cyclin D* and *E2F1*. A-C.Effect of *LEF1* and its mutations on promoter activity of *c-MYC* (A), *Cyclin D1* (B) and *E2F1* (C). D.Comparison of *c-MYC* and *CyclinD1* mRNA expression in Nalm6 cells expressing *LEF1*-WT, *LEF1*-K86E, *LEF1*-P106L and vector only control.* *P*<0.05; **P<0.01.

**Fig 6:**
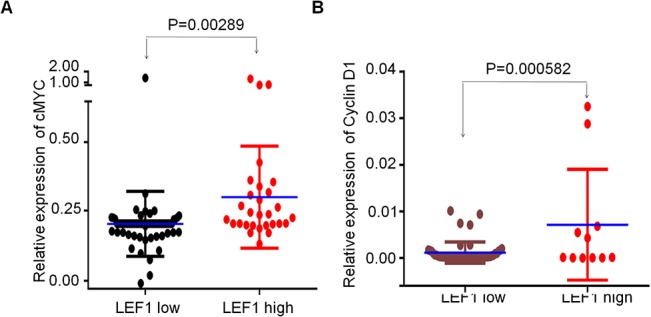
Difference of *c-MYC* and *Cyclin D1* expression in ALL patients. A-B The *c-MYC* (A) and *Cyclin D1* (B) expression in ALL patients with *LEF1* high or low expression

To understand the underlying mechanism of *LEF1* and its mutations on proliferation stimulation, we explored their effect on transcription of *LEF1* targets by luciferase reporter assay. We found that *LEF1*-WT obviously increased the promoter activity of *c-MYC* and *Cyclin D1* compared to vector only. Furthermore, both mutants could significantly increase the promoter activity of *c-MYC* and *Cyclin D1* compared to that of *LEF1*-WT. We also examined the expression of *c-MYC* and *Cyclin D1* in Nalm6 cells expressing the *LEF1*-WT or its mutants by qPCR. We found that the expression of *c-MYC* and *Cyclin D1* was significantly increased in *LEF1*-WT cells and the two mutant cells (Fig 5). More importantly, we observed *c-MYC* and *Cyclin D1* expression in patients with *LEF1* high expression was significantly higher than that of low expression (Fig 6). Moreover, the 2 patients with *LEF1* mutations had high *c-MYC* and *Cyclin D1* expression (no samples available for the other two patients with the mutations) (data not shown). These data indicated that high expression of *LEF1* and possibly its mutants (K86E and P106L) promote cell proliferation by increasing the expression of *c-MYC* and *Cyclin D1*.

## Discussion

Around half of adult ALL patients, including patients with standard risk without known poor-risk features, still develop relapse, which is associated with an extremely poor survival rate of < 10% although the treatment has dramatically improved. Reduction of relapse rate is therefore the main aim of treatment optimization in adult ALL. Many factors are responsible for ALL relapse. High expression of key genes in multioncogenic pathways and mutations in the essential genes involved in lymphocyte development are among the high-risk factors for ALL relapse and treatment response. Here we observed that high *LEF1* expression was significantly correlated with high-risk B-ALL and T-ALL to some extent, and identified two novel *LEF1* mutations which may be associated with poor outcome. We found the novel *LEF1* mutations could promote the cell proliferation of leukemia cells by regulation of gene expression of *LEF1* targets: *c-MYC* and *Cyclin D1*. Our findings suggest high *LEF1* expression and mutations play important roles in development of high-risk ALL.

Thus far, there have been several reports about the *LEF1* expression in leukemia and its impact on outcome of the patients. LEF1 expression was reported to be elevated in adult ALL[6], CLL/lymphoma [24–29] and AML[4,8,10,30]. High *LEF1* expression is associated with poor prognosis in adult B precursor ALL and CLL [6,24,26,29,31]. Kuhnl A and his colleagues reported that overexpressed *LEF1* was observed in 71/282 patients with ALL[6]. They also found that the patients with high *LEF1* expression had a significant shorter relapse-free survival (RFS) and the high *LEF1* expression was also associated with inferior RFS in standard-risk patients[6]. Gutierrez A Jr et al reported the aberrant protein expression of *LEF1* specifically in CLL not normal B cells and they also identified *LEF1* expression in CD19(+)/CD5(+) cells obtained from patients with monoclonal B-cell lymphocytosis, suggesting a role for *LEF1* early in CLL leukemogenesis [26]. Moreover, an obvious downregulation of *LEF1* has been associated with disease progression in myelodysplastic syndrome [32]. High *LEF1* expression has also been reported as an unfavorable prognostic marker in cytogenetically normal AML [10,30] and in adult de novo APL [8]. Recently, it was reported that BCR-ABL regulates IRES-mediated translation of *LEF1* in CML [33]. Our data also showed that *LEF1* high expression is significantly associated with high WBC in B-ALL and T-ALL; also *LEF1* promoted the cell proliferation of B-ALL and T-ALL cells. *LEF1* high expression is significantly associated with shorter EFS in B-ALL patients. Our findings underscore the pro-survival and oncogenic effect of *LEF1* in acute leukemia particularly B-ALL, revealing high *LEF1* expression may link to high-risk ALL.

We also performed mutational analysis in 131 ALL patients and detected 2 novel mutations in 4 patients, which were not reported previously. The correlation of mutations with survival is not clear because of the limited patients’ number. However we did observe that patients with *LEF1* mutations had high-risk factors such as high WBC, complex karyotype, and relapsed in 3 months. The frequency of the mutation in this cohort of ALL is 3.1%, which is higher than reported in pre-B ALL (1.6%) and lower than that in T-ALL. We did not find any other single nucleotide polymorphisms or mutations in our cohort. Our data also showed that the mutations had stronger effects on proliferation stimulation and target genes’ expression than *LEF1* wild- type, which indicated that the mutant has an oncogenic effect in the patients even when its expression is low.

Gutierrez et al also found a microdeletion mutation in the N-terminal of *LEF1* in 11% of pediatric T-ALL [7]. We did not find any deletions in the 131 ALL patients. Functional analysis showed the *LEF1* mutations we identified are gain-of-function mutations, which is different from Gutierren’s report. We observed that *LEF1* high expression is significantly correlated with *Notch1* and *JAK1* mutations in T-ALL patients. These findings indicate that *LEF1* high expression together with the genetic alterations of other key molecules may contribute to the formation of adult high-risk T-ALL.

*LEF1* interacts with nuclear catenin in the Wnt signaling pathway [34]. As initiation step of Wnt signaling, β-catenin is stabilized in the cytoplasm and subsequent accumulated in the nucleus, where β-catenin interacts with TCF/LEF DNA binding effectors to modulate the transcription of numerous target genes which are involved in cell cycle progression, extracellular matrix remodeling, cell adhesion, and cell differentiation. The *c-MYC* and *Cylclin D1* are reported *LEF1* targets and *LEF1* activates *c-MYC* and *CyclinD1*. Our data showed wild-type *LEF1* promotes the transcription and increased mRNA level of *c-MYC* and *Cyclin D1*, indicating *LEF1* promotes cell proliferation in ALL cells by regulation of gene expression of those targets. The two *LEF1* mutants could further increase the transcription of *c-MYC* and *Cyclin D1* over *LEF1* wild-type, indicating that the *LEF1* mutations are gain-of-function mutants and promote cell proliferation by regulation of *LEF1* gene targets.

## Conclusions

We examined the *LEF1* expression in adult ALL patients and identified 2 novel *LEF1* mutations. Our finding indicated high *LEF1* expression and *LEF1* mutation are associated with high-risk leukemia and our results also indicated that the high expression and/or gain-of-function mutations account for the oncogenic effect of *LEF1* in ALL.
