# Correction: Characterization of *LEF1* High Expression and Novel Mutations in Adult Acute Lymphoblastic Leukemia

**DOI:** 10.1371/journal.pone.0307986

**Published:** 2024-07-24

**Authors:** Xing Guo, Run Zhang, Juan Liu, Min Li, Chunhua Song, Sinisa Dovat, Jianyong Li, Zheng Ge

Fig 4 is uploaded incorrectly. Please view Fig 4 here.

**Fig 4:**
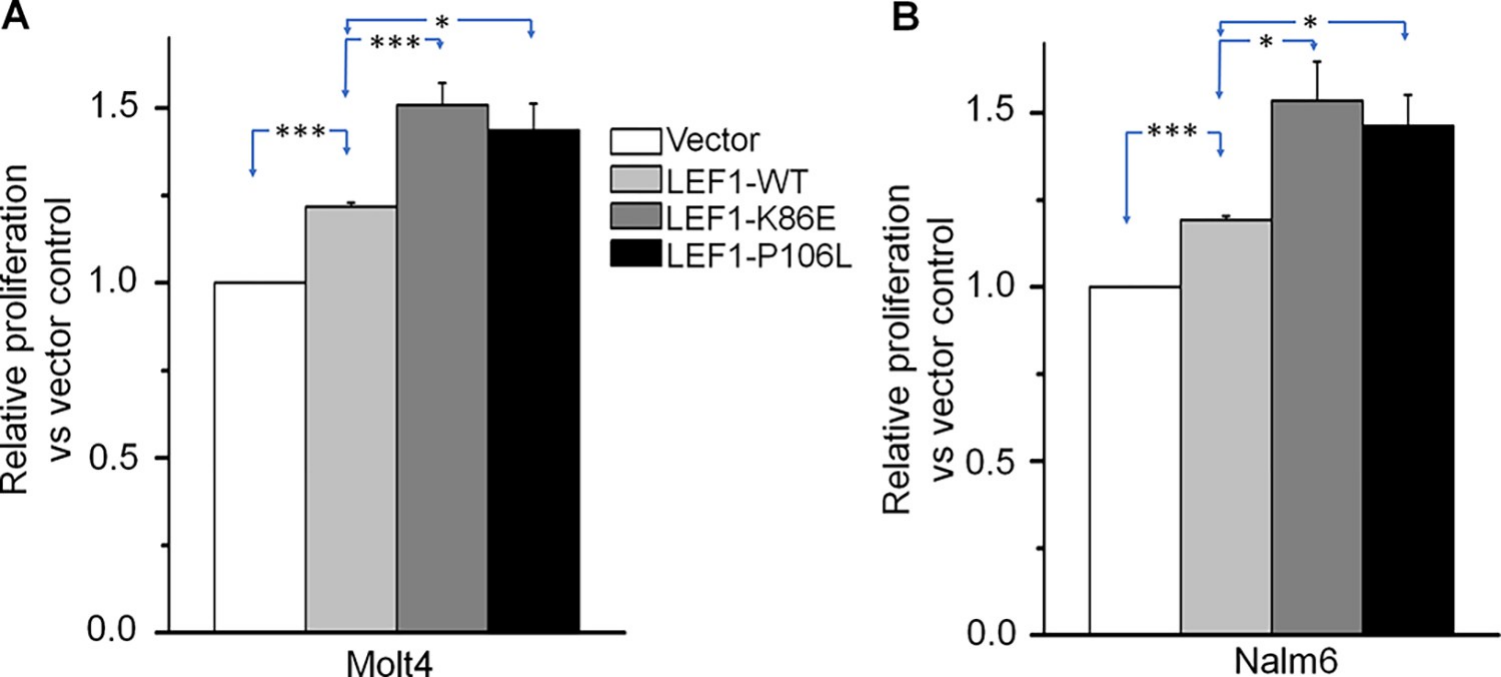
*LEF1* mutants promote cell proliferation of ALL cells. A-B.*LEF1* wild-type (*LEF1*-WT) and its mutants (*LEF1*-K86E and *LEF1*-P106L) were expressed in Molt4 (A) and Nalm6 (B) cells. * *P*<0.05; **P<0.01; ***P<0.001.
